# Supplementary figures and images for: Healthcare in England was affected by the COVID-19 pandemic across the pancreatic cancer pathway: A cohort study using OpenSAFELY-TPP
Source: eLife. 2023 Aug 10;12:e85332. doi: 10.7554/eLife.85332 (PMC10414967; doi:10.7554/eLife.85332)

**Supplement Figure 1. Study flowchart**

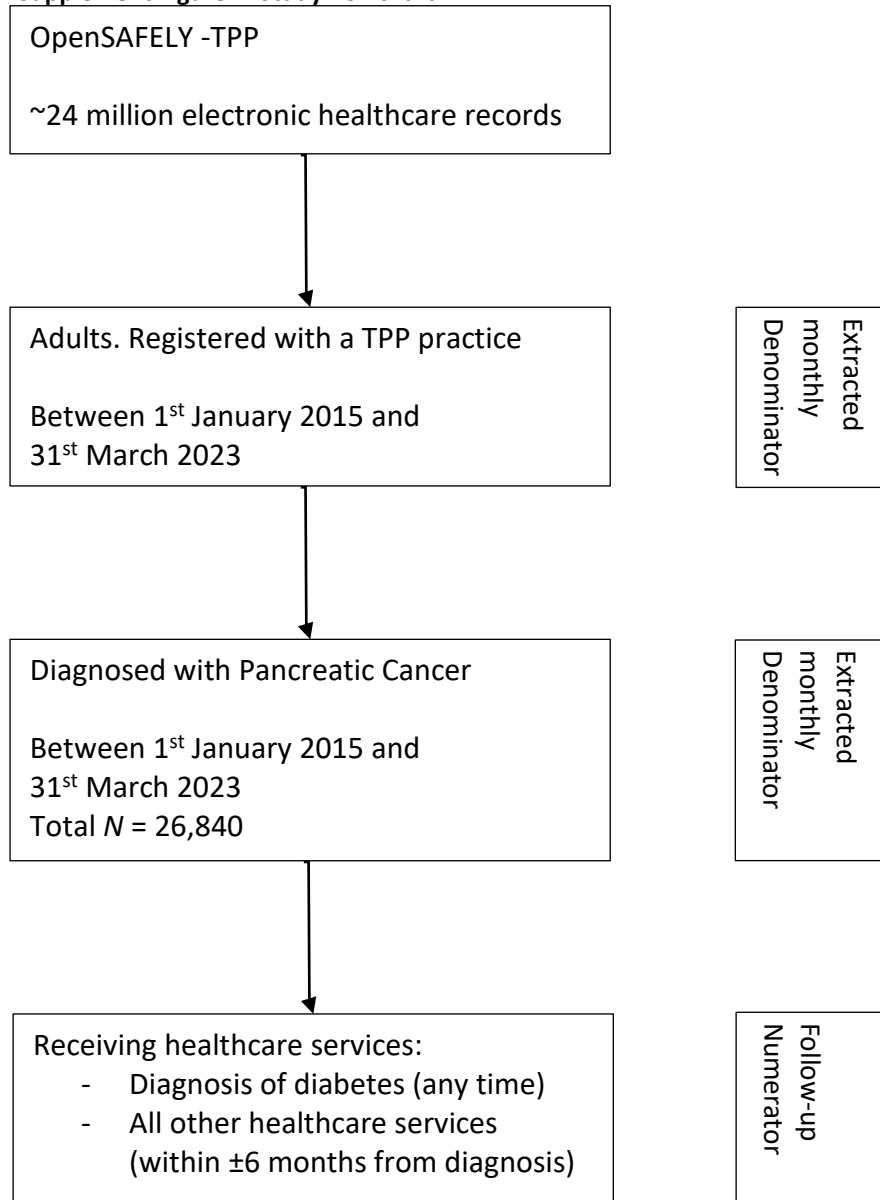

Supplement: Supplementary file 1. [file elife-85332-supp1.pdf]
